# Supplementary material for: Dorsal hippocampus mediates light–tone associations in male mice
Source: eLife. 2025 Dec 3;14:RP105863. doi: 10.7554/eLife.105863 (PMC12674615; doi:10.7554/eLife.105863)
Supplement: Supplementary file 1. [file elife-105863-supp1.docx]

**Supplementary Material for**

**Dorsal hippocampus mediates light-tone associations in male mice**

Julia S. Pinho^1,2^, Carla Ramon-Duaso^1^, Irene Manzanares-Sierra^1^, Arnau Busquets-García^1^

^1^ Cell-Type Mechanisms in Normal and Pathological Behavior Research Group, Neuroscience Research Program, Hospital del Mar Research Institute, Barcelona, Spain

^2^ Current address: Gulbenkian Institute for Molecular Medicine, Oeiras, Portugal

**Content:**

**Supplementary Tables 1-2**

**Table A.** Statistical analysis. Related to Main Figures 1-4

| Fig. | Condition | n (per group) | Statistical method  (interaction main effect) | Factors analysed | Normality and homoscedasticity | Planned Comparisons or Posthoc | P-value |
| --- | --- | --- | --- | --- | --- | --- | --- |
| 1C | Mediated | 12 | GLM=-5.36, p_value=0.013 | Freezing,experiment(paired, unpaired, no-shock),  Off vs On | No | Paired Off vs On | 0,004 |
|  |  |  |  |  |  | Unpaired Off vs On | 0,083 |
|  |  |  |  |  |  | No-shock Off vs On | 0,044 |
|  |  |  |  |  |  | Off Paired vs Unpaired | 1,000 |
|  |  |  |  |  |  | Off Paired vs No-shock | 0,046 |
|  |  |  |  |  |  | Off Unpaired vs No-shock | <0,001 |
|  |  |  |  |  |  | On Paired vs Unpaired | 1,000 |
|  |  |  |  |  |  | On Paired vs No-shock | 0,001 |
|  |  |  |  |  |  | On Unpaired vs No-shock | <0,001 |
| 1E | Direct | 12 | GLM=-18.09, p_value=0.000 | Freezing,experiment(paired, unpaired, no-shock),  Off vs On | No | Paired Off vs On | 0,004 |
|  |  |  |  |  |  | Unpaired Off vs On | 0,004 |
|  |  |  |  |  |  | No-shock Off vs On | 0,004 |
|  |  |  |  |  |  | Off Paired vs Unpaired | 0,065 |
|  |  |  |  |  |  | Off Paired vs No-shock | 0,065 |
|  |  |  |  |  |  | Off Unpaired vs No-shock | 0,001 |
|  |  |  |  |  |  | On Paired vs Unpaired | 1,000 |
|  |  |  |  |  |  | On Paired vs No-shock | <0,001 |
|  |  |  |  |  |  | On Unpaired vs No-shock | <0,001 |
| 2B left | Dorsal hippocampus | 7 | Wilcoxon with zero method | Calcium signal (RCaMP or GCaMP), time | No | Gcamp before vs after | 0.0156 |
|  |  |  |  |  |  | Rcamp before vs after | 0.0312 |
| 2C left | Ventral hippocampus | 8 | Wilcoxon with zero method | Calcium signal (RCaMP or GCaMP), time | No | Gcamp before vs after | 0.0156 |
|  |  |  |  |  |  | Rcamp before vs after | 0.0078 |
| 2D | Maximum delta df | Same as 2B and 2C | Wilcoxon with zero method | Calcium signal (RCaMP or GCaMP), time | No | Same as 2B and 2C | |
| 3B | Dorsal hippocampus | 6 | Wilcoxon with zero method | Calcium signal (GCaMP), time | No | Gcamp before vs after | |
| 3C | Ventral hippocampus | 6 | Wilcoxon with zero method | Calcium signal (GCaMP), time | No | Gcamp before vs after | |
| 3D | Maximum delta df | Same as 3B and 3C | Wilcoxon with zero method | Calcium signal (GCaMP), time | No | Same as 3B and 3C | |
| 4B | Mediated | 11-19 | GLM=-1.61, p_value=0.505 | Freezing,dorsal_inhibition(controls, DPC, DPT),  Off vs On | No | Controls_d Off vs On | <0,001 |
|  |  |  |  |  |  | DPCd Off vs On | 0,756 |
|  |  |  |  |  |  | DPTd Off vs On | 0,035 |
|  |  |  |  |  |  | Off Controls_d vs DPCd | 0,221 |
|  |  |  |  |  |  | Off Controls_d vs DPTd | 1,000 |
|  |  |  |  |  |  | Off DPCd vs DPTd | 1,000 |
|  |  |  |  |  |  | On Controls_d vs DPCd | 1,000 |
|  |  |  |  |  |  | On Controls_d vs DPTd | 1,000 |
|  |  |  |  |  |  | On DPCd vs DPTd | 1,000 |
| 4C | Mediated | 11-21 | GLM=1.75, p_value=0.539 | Freezing, ventral_inhibition (controls, DPC, DPT),  Off vs On | No | Controls_v Off vs On | 0,005 |
|  |  |  |  |  |  | DPCv Off vs On | 0,018 |
|  |  |  |  |  |  | DPTv Off vs On | 0,018 |
|  |  |  |  |  |  | Off Controls_v vs DPCv | 1,000 |
|  |  |  |  |  |  | Off Controls_v vs DPTv | 1,000 |
|  |  |  |  |  |  | Off DPCv vs DPTv | 1,000 |
|  |  |  |  |  |  | On Controls_v vs DPCv | 1,000 |
|  |  |  |  |  |  | On Controls_v vs DPTv | 1,000 |
|  |  |  |  |  |  | On DPCv vs DPTv | 1,000 |
| 4D | Direct | 11-19 | GLM=3.15, p_value=0.398 | Freezing,dorsal_inhibition(controls, DPC, DPT),  Off vs On | No | Controls_d Off vs On | <0,001 |
|  |  |  |  |  |  | DPCd Off vs On | 0,018 |
|  |  |  |  |  |  | DPTd Off vs On | 0,035 |
|  |  |  |  |  |  | Off Controls_d vs DPCd | 1,000 |
|  |  |  |  |  |  | Off Controls_d vs DPTd | 1,000 |
|  |  |  |  |  |  | Off DPCd vs DPTd | 1,000 |
|  |  |  |  |  |  | On Controls_d vs DPCd | 1,000 |
|  |  |  |  |  |  | On Controls_d vs DPTd | 1,000 |
|  |  |  |  |  |  | On DPCd vs DPTd | 1,000 |
| 4E | Direct | 11-21 | GLM=2.46, p_value=0.421 | Freezing, ventral_inhibition (controls, DPC, DPT),  Off vs On | No | Controls_v Off vs On | <0,001 |
|  |  |  |  |  |  | DPCv Off vs On | 0,018 |
|  |  |  |  |  |  | DPTv Off vs On | 0,035 |
|  |  |  |  |  |  | Off Controls_v vs DPCv | 1,000 |
|  |  |  |  |  |  | Off Controls_v vs DPTv | 1,000 |
|  |  |  |  |  |  | Off DPCv vs DPTv | 1,000 |
|  |  |  |  |  |  | On Controls_v vs DPCv | 1,000 |
|  |  |  |  |  |  | On Controls_v vs DPTv | 1,000 |
|  |  |  |  |  |  | On DPCv vs DPTv | 1,000 |

| Fig.  **Table B.** Statistical analysis. Related to Figures Supplements | Condition | n (per group) | Analysis | Factors analysed | Normality and homoscedasticity | Comparison | | P-value |
| --- | --- | --- | --- | --- | --- | --- | --- | --- |
| Fig. 1-fig. suppl 1 upper | All phases | 12 | Spearman correlation (Eztrack: R_males_=0.91, R_females_=0.86, Manual: R_males_=0.96, R_females_=0.95) | Freezing, Sex, method | No | Deeplacut vs Eztrack males | | 0.0001 |
|  |  |  |  |  |  | Deeplacut manual females | | 0.0001 |
| Fig. 1-fig. suppl 1 bottom | Probe test | 12 | Spearman correlation (Eztrack: R_males_=0.94, R_females_=0.88, Manual: R_males_=0.99, R_females_=0.97) | Freezing, Sex, method | No | Deeplacut vs Eztrack males | | 0.0001 |
|  |  |  |  |  |  | Deeplacut manual females | | 0.0001 |
| Fig. 1-fig. suppl 2C | Mediated | 24 | GLM=-7.06, p_value=0.000 | Freezing,experiment(paired, unpaired, no-shock),  Off vs On, females | No | Paired Off vs On | | <0,001 |
|  |  |  |  |  |  | Unpaired Off vs On | | <0,004 |
|  |  |  |  |  |  | No-shock Off vs On | | 1.000 |
|  |  |  |  |  |  | Off Paired vs Unpaired | | 1.000 |
|  |  |  |  |  |  | Off Paired vs No-shock | | 1.000 |
|  |  |  |  |  |  | Off Unpaired vs No-shock | | 1.000 |
|  |  |  |  |  |  | On Paired vs Unpaired | | 0.351 |
|  |  |  |  |  |  | On Paired vs No-shock | | <0,001 |
|  |  |  |  |  |  | On Unpaired vs No-shock | | 1.000 |
| Fig. 1-fig. suppl 2E | Direct | 24 | GLM=-14.23, p_value=0.000 | Freezing,experiment(paired, unpaired, no-shock),  Off vs On | No | Paired Off vs On | | <0,001 |
|  |  |  |  |  |  | Unpaired Off vs On | | 0,004 |
|  |  |  |  |  |  | No-shock Off vs On | | 0,242 |
|  |  |  |  |  |  | Off Paired vs Unpaired | | 1,000 |
|  |  |  |  |  |  | Off Paired vs No-shock | | 1,000 |
|  |  |  |  |  |  | Off Unpaired vs No-shock | | 0,545 |
|  |  |  |  |  |  | On Paired vs Unpaired | | 1,000 |
|  |  |  |  |  |  | On Paired vs No-shock | | <0,001 |
|  |  |  |  |  |  | On Unpaired vs No-shock | | 0,001 |
| Fig. 1-fig. suppl 3A | Mediated, Direct | 12 | Spearman correlation (Mediated vs Direct: R_males_=0.70) | Freezing, Sex | No | 0.011 | | |
| Fig. 1-fig. suppl 3B | Mediated, Direct | 24 | Spearman correlation (Mediated vs Direct: R_females_=0.02) | Freezing, Sex | No | 0.920 | | |
| Fig. 2-fig. suppl 1B | Dorsal hippocampus | 7 | Wilcoxon with zero method | Calcium signal (Rcamp or Gcamp), time | No | Gcamp before vs after | | 0.015625 |
|  |  |  |  |  |  | Rcamp before vs after | | 0.03125 |
| Fig. 2-fig. suppl 1C left | Ventral hippocampus | 8 | Wilcoxon with zero method | Calcium signal (Rcamp or Gcamp), time | No | Gcamp before vs after | | 0.015625 |
|  |  |  |  |  |  | Rcamp before vs after | | 0.0234375 |
| Fig. 2-fig. suppl 1D | Maximum delta df | Same as S4B and C | Wilcoxon with zero method | Calcium signal (Rcamp or Gcamp), time, dorsal and ventral hippocampus | No | Same as S4B and C | | |
| Fig. 2-fig. suppl 2B | Dorsal hippocampus | 7 | Wilcoxon with zero method | Calcium signal (Rcamp or Gcamp), time | No | Gcamp before vs after | 0.015625 | |
|  |  |  |  |  |  | Rcamp before vs after | 0.03125 | |
| Fig. 2-fig. suppl 2C | Ventral hippocampus | 8 | Wilcoxon with zero method | Calcium signal (Rcamp or Gcamp), time | No | Gcamp before vs after | 0.015625 | |
|  |  |  |  |  |  | Rcamp before vs after | 0.0234375 | |
| Fig. 2-fig. suppl 2D | Maximum delta df | Same as S3B and C | Wilcoxon with zero method | Calcium signal (Rcamp or Gcamp), time, dorsal and ventral hippocampus | No | Same as S3B and C | | |
| Fig. 2-fig. suppl 3B | Dorsal hippocampus | 7 | Wilcoxon with zero method | Calcium signal (Rcamp or Gcamp), time | No | Gcamp before vs after | 0.015625 | |
|  |  |  |  |  |  | Rcamp before vs after | 0.03125 | |
| Fig. 2-fig. suppl 3C | Ventral hippocampus | 8 | Wilcoxon with zero method | Calcium signal (Rcamp or Gcamp), time | No | Gcamp before vs after | 0.015625 | |
|  |  |  |  |  |  | Rcamp before vs after | 0.0078125 | |
| Fig. 2-fig. suppl 3D | Maximum delta df | Same as S5B and C | Wilcoxon with zero method | Calcium signal (Rcamp or Gcamp), time, dorsal and ventral hippocampus | No | Same as S4B and C | | |
| Fig. 4-fig. suppl 1A | Mediated | 21 | Kruskall-Walis=4.73 | Freezing, dorsal controls groups (CS, CJ60, DS) | No | 0.094 | | |
| Fig. 4-fig. suppl 1B | Direct | 21 | Kruskall-Walis=0.99 | Freezing, dorsal controls groups (CS, CJ60, DS) | No | 0.610 | | |
| Fig. 4-fig. suppl 1C | Mediated | 19 | Kruskall-Walis=0.58 | Freezing, ventral controls groups (CS, CJ60, DS) | No | 0.748 | | |
| S Fig. 4-fig. suppl 1D | Direct | 19 | Kruskall-Walis=1.99 | Freezing, ventral controls groups (CS, CJ60, DS) | No | 0.370 | | |
| Fig. 4-fig. suppl 1^E^ | Calcium frequency | 4 | Wilcoxon test | Basal vs j60 | No | 0.25 | | |
| Fig. 4-fig. suppl 1F | Calcium frequency | 4 | Wilcoxon test | Basal vs j60 | No | 0.25 | | |
| Fig. 4-fig. suppl 1G | Freezing | 22 | Mixed-effects analysis followed by Tuke’s multiple comparisons test | Freezing and drug | No | Saline : Off vs On | 0.0006 | |
|  |  |  |  |  | No | J60: Off vs On | 0.012 | |
|  |  |  |  |  | No | On : saline vs J60 | 0.024 | |
| Fig. 4-fig. suppl 2B | Calcium frequency | 4 | Wilcoxon test | Basal vs j60 | No | 0.187 | | |
| Fig. 4-fig. suppl 2C | Calcium frequency | 4 | Wilcoxon test | Basal vs j60 | No | 0.625 | | |
| Fig. 4-fig. suppl 2D | Mediated | 11 | GLM= -16.57,  p value=0.001 | Freezing | No | Off vs On | | 0.049 |
| Fig. 4-fig. suppl 2D | Direct | 11 | GLM= -16.57,  p value=0.001 | Freezing | No | Off vs On | | 0.00 |
